# T1-weighted MRI as a substitute to CT for skull aberration correction in transcranial focused ultrasound: i*n vivo f*easibility and i*n vitro c*omparison on human calvaria

**DOI:** 10.1186/2050-5736-3-S1-P21

**Published:** 2015-06-30

**Authors:** Apoorva Iyer, Olivia Hatch, Nicholas Tustison, James Patrie, Wenjun Xin, Matt Eames, Suna Sumer, Benison Lau, Alan Cupino, John Snell, Arik Hananel, Neal Kassell, Jean-Francois Aubry, Max Wintermark

**Affiliations:** 1 Focused Ultrasound Foundation, Charlottesville, Virginia, United States; 2 University of Virginia, Charlottesville, Virginia, United States; 3 Institut Langevin, Paris, France

## Background/introduction

CT scans have been used to refocus ultrasound for clinical transcranial MRI-guided focused ultrasound (TcMRgFUS). The refocusing of the ultrasound arrays is a crucial step in TcMRgFUS therapy because skulls distort sound beams due to the ununiformed shape and composition of the bone. CT scan based algorithms have been developed to estimate the phase shifts induced by the bone. The corresponding phase conjugated signals are then emitted by ultrasound arrays to be refocused on brain targets through the skull. However, CT scans elongate the TcMRgFUS treatment process and expose patients to ionizing radiation. This study tested three different MRI pulse sequences (T1, T2, and proton density) against CT to determine if MRI data could be a viable alternative to CT for planning ultrasound refocusing in TcMRgFUS.

## Methods

Ten adult patients who had CT scans showing an intact skull and were scheduled for an MRI of the brain were enrolled in this study and had three MR scans: 3DT weighted 3D volume interpolated breath hold examination (VIBE), proton density weighted 3D Sampling Perfection with Application Optimized Contrast using different flip angle Evolutions (SPACE), and 3D true Fast Imaging with Steady State Precession T2-weighted imaging (FISP). Template masks were created from the MRI data and Bayesian segmentation was then applied to calculate total skull thickness and individual thicknesses of the three skull layers (inner table, diploe and outer table). The virtual average CT density was derived from the average MRI intensity. A first training-dataset was compiled to create regression predictions which were then applied to a second validation-dataset. The discrepancy in measurements of total skull thickness between the MRI modalities and the CT modalities was examined and the optimal MRI sequence was identified. Phase shifts were calculated by importing standard and virtual CT data into the ExAblate© Neuro system operated at 720kHz and formatting the virtual CT data header to mimic real CT data. As further validation, a phantom study was performed, in which a degassed human skull was embedded in a mannequin head and filled with tissue-like hydrogel. The standard and virtual CT data were imported into the software and phase correction was used to sonicate and induce a thermal rise at the desired focal point in the phantom.

## Results and conclusions

The T1 sequence gave values closest to those of the CT reference for total skull thickness (mean discrepancy = 0.025 + 0.112, p = 0.825). T1 MRI sequence was found to systematically overestimate CT skull thickness for thicker skulls and underestimate for thinner skulls. The accuracy of the predictions of CT inner, middle, and outer skull layers ranged from 0.42 to 0.57 mm. For the phantom data set, the mean absolute difference between the standard CT and MRI derived CT was 0.8 + 0.6 rad with mean 7 + 4% drop in temperature elevation. These results indicate that MRI based phase correction in TcMRgFUS is a viable alternative to CT based refocusing.

